# Genome Sequence Variability Predicts Drug Precautions and Withdrawals from the Market

**DOI:** 10.1371/journal.pone.0162135

**Published:** 2016-09-30

**Authors:** Kye Hwa Lee, Su Youn Baik, Soo Youn Lee, Chan Hee Park, Paul J. Park, Ju Han Kim

**Affiliations:** 1 Seoul National University Biomedical Informatics (SNUBI), Division of Biomedical Informatics, Seoul National University College of Medicine, Seoul 110799, Korea; 2 Biomedical Informatics Training and Education Center (BITEC), Seoul National University Hospital, Seoul 110744, Korea; 3 Department of Physiology and Cell Biology, University of Nevada School of Medicine, Reno, Nevada, United States of America; National Chiao Tung University College of Biological Science and Technology, TAIWAN

## Abstract

Despite substantial premarket efforts, a significant portion of approved drugs has been withdrawn from the market for safety reasons. The deleterious impact of nonsynonymous substitutions predicted by the SIFT algorithm on structure and function of drug-related proteins was evaluated for 2504 personal genomes. Both withdrawn (*n* = 154) and precautionary (Beers criteria (*n* = 90), and US FDA pharmacogenomic biomarkers (*n* = 96)) drugs showed significantly lower genomic deleteriousness scores (*P* < 0.001) compared to others (*n* = 752). Furthermore, the rates of drug withdrawals and precautions correlated significantly with the deleteriousness scores of the drugs (*P* < 0.01); this trend was confirmed for all drugs included in the withdrawal and precaution lists by the United Nations, European Medicines Agency, DrugBank, Beers criteria, and US FDA. Our findings suggest that the person-to-person genome sequence variability is a strong independent predictor of drug withdrawals and precautions. We propose novel measures of drug safety based on personal genome sequence analysis.

## Introduction

The person-to-person variability in drug response is a major challenge for current clinical practice, drug development, and drug regulation.[1] A drug with proven clinical efficacy in some patients often fails to work in others and may even cause serious side effects, including death.[1–3] The incidence of severe adverse drug reactions (ADRs) has been estimated at 6.2–6.7% in hospitalized patients, and more than 2 million ADR cases occur annually in the United States (US), including 100,000 deaths.[3,4] As a result, many drugs causing unexpected severe ADRs are eventually withdrawn from the market.

The impact and cost burden of pharmaceutical market withdrawals are enormous. Of the 548 drugs that were newly approved by the US Food and Drug Administration (FDA) between 1975 and 1999, 56 (10.2%) received a boxed warning or were eventually withdrawn from the market.[5,6] Twenty (3.8%) of the 528 drugs approved between 1990 and 2009 in Canada were withdrawn for safety reasons, and the percentage of drug removals from the market did not change significantly during that period indicating that there was also no change in the effectiveness of the drug review system.[7] Despite substantial premarket evaluation efforts, including long and costly clinical trials, postmarket drug withdrawals are generally not preventable by the currently available means.[8,9]

The variability of drug responses among different individuals, which makes long-term predictability of drug performance difficult, may be explained by the genotypic diversity in the population. Advancements in pharmacogenomics now provides the information about the effects of genetic variations on individual drug responses,[10] which is currently listed on the labels of approximately 150 drugs that have been approved by the US FDA.[11] This accumulation of genetic data relevant to drug response is a significant step towards realization of *personalized medicine*. Population-based genome-wide observational research, such as Genome-Wide Association Studies (GWAS), is currently one of the most powerful tools for investigating the relationship between genotypic variability and drug responses. However, the current population-based approach in an affected-versus-nonaffected case-control setting is inherently limited because the genotypes, drugs, and their associations are too numerous to be reliably tested in a foreseeable future. Thus, a large number of commonly used drugs are not examined by GWAS.

Genome sequencing technology has revealed hundreds of genetic variants that are predictive of loss of function (LoF) in protein-coding genes.[12] The genes related to drug pharmacokinetics and pharmacodynamics (PK/PD) have many LoF variants, and their prevalence shows significant person-to-person variability.[13] The strikingly large number of deleterious genetic variants even in apparently healthy subjects and their uneven distribution across individual genomes may explain the unusual responses to certain drugs in small subpopulations.[14] Clinical trials are generally conducted in groups that are homogeneous in age, gender, and/or ethnicity, and the results are then extrapolated to the general population.[15] However, the extrapolation may not be applicable to certain genetically differentiated subpopulations, which may not have been significantly represented during phase-III clinical trials. The significant inclusion of the susceptible subpopulations following premarket approval of a drug by regulatory authorities may then result in unexpected ADRs that may ultimately lead to its withdrawal from the pharmaceutical market. Therefore, we hypothesized that the person-to-person variability in the distribution of deleterious variants of PK/PD genes in different ethnic groups will reveal subpopulations predisposed to severe ADRs, which may subsequently result in drug removal from the market.

Our findings suggest that the person-to-person genetic variability is a strong independent predictor of drug withdrawals for safety reasons. Therefore, we propose a method to identify individuals (or subpopulations) with increased vulnerability to side effects from use of specific drugs and suggest novel measures to ensure drug safety based on personal genome sequence analysis, which can improve drug development, use, and regulations.

## Materials and Methods

### Drugs, Genes, and Genomes

**Fig 1:**
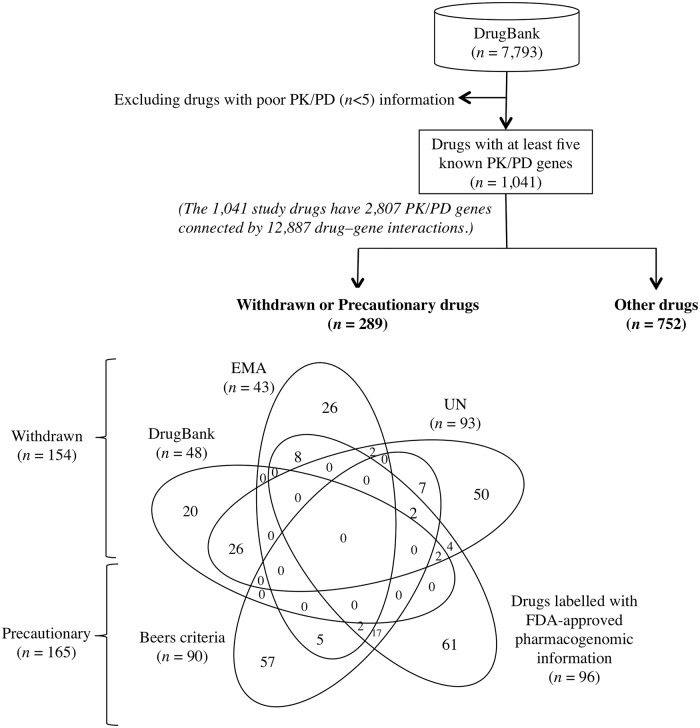
Inclusion criteria and distribution of withdrawn, precautionary, and other drugs. In total, 1041 drugs from the DrugBank with at least five identified genes involved in drug pharmacokinetics (PK) and pharmacodynamics (PD) were included in the study. A comprehensive list of withdrawn drugs (*n* = 154) was obtained by reviewing three resources: 1) the 8th, 10th, 12th, and 14th Issues of the ‘Consolidated List of Products Whose Consumption and/or Sale Have Been Banned, Withdrawn, Severely Restricted, or Not Approved by Governments: Pharmaceuticals’ published by the United Nations [18–21]; 2) applications for withdrawal from European Medicines Agency (EMA)[22]; and 3) the DrugBank annotations for withdrawals. Precautionary drugs (*n* = 165) were identified based on the Beers criteria [23] and US FDA table of pharmacogenomics biomarkers [11]. The remaining 752 drugs were classified as other drugs.

The DrugBank database is a bioinformatics resource that provides detailed drug information, including chemical structure, pharmacological mechanism, drug targets, metabolic enzymes, carriers, and transporters.[16] Among the 7793 drugs in the DrugBank, 5099 drugs had at least one identified PK/PD genes (accessed September 4, 2015 at http://www.drugbank.ca/), of which 1041 drugs having five or more PK/PD genes were included in the following analysis (Fig 1) by excluding 4058 drugs having less than five PK/PD genes. In our study, we analyzed the 1041 drugs with five or more PK/PD genes and 2807 PK/PD genes known to be involved in drug responses via 12,887 drug-gene interactions (Fig 1 and S1 File). We downloaded 2504 publicly available personal genomes from the 1000 Genomes Project, which provided unbiased data on the genetic variability within 26 ethnic subgroups from Europe, East Asia, South Asia, Africa, and the Americas [17] (accessed September 4, 2015 at http://www.1000genomes.org/).

### Selection of Precautionary and Market-withdrawn Drugs

In order to create a comprehensive list of withdrawn drugs, we performed an in-depth review of the following publically available resources: 1) the 8th, 10th, 12th, and 14th Issues of the ‘Consolidated List of Products Whose Consumption and/or Sale Have Been Banned, Withdrawn, Severely Restricted, or Not Approved by Governments: Pharmaceuticals’ published by the United Nations (UN Consolidated Lists) since 2003,[18–21] which are the most comprehensive lists of withdrawn and severely restricted drugs produced by at least one of 94 governments, 2) drug applications withdrawn by the Committee for Medicinal Products for Human Use (CHMP) at the European Medicines Agency (EMA),[22] and 3) DrugBank annotations for pharmaceutical market withdrawals.[16] (S2 File)

In our study, a drug was defined as “withdrawn” if it had been removed, banned, or disapproved by at least one country for any reason. We manually selected 350 drugs that were withdrawn by at least one country according to the UN Consolidated Lists [18–21] and 174 drugs that were withdrawn from January 2006 to September 2015 according to the EMA.[22] Furthermore, the DrugBank annotated 181 drugs as withdrawn. For our analysis, we also selected “precautionary” drugs by applying the Beers criteria (*n* = 137)[23] and US FDA pharmacogenomic biomarker information in drug labels (*n* = 148).[11] The Beers criteria for Potentially Inappropriate Medication Use in Older Adults, which was initially published in 1991 [24] and most recently revised by the American Geriatric Society in 2012,[25] is widely used in clinical care to prevent ADRs in the elderly population. Fig 1 shows that among the 1041 drugs with at least five PK/PD genes, 289 were classified as withdrawn or precautionary (*n* = 165). “Other” drugs were not included in the list of precautionary drugs and also not withdrawn by any of the available resources (n = 752). Among the 154 withdrawn drugs, 16 and 18 were also redundantly classified as precautionary according to the Beers criteria (*n* = 90) and US FDA pharmacogenomic biomarker information (*n* = 96), respectively (Fig 1).

### Deleteriousness Scores for Genes, Variants, Drugs, and Populations

The genes encoding for drug targets, related metabolic enzymes, and drug transporters affect an individual’s response to a drug. Furthermore, previous studies have demonstrated that the impact of nonsynonymous substitutions on protein structure and function can be reliably predicted by applying straightforward empirical rules using specific algorithms such as SIFT,[26] PolyPhen-2,[27] MutationAssessor,[28] CADD,[29] and Condel.[30]

**Fig 2:**
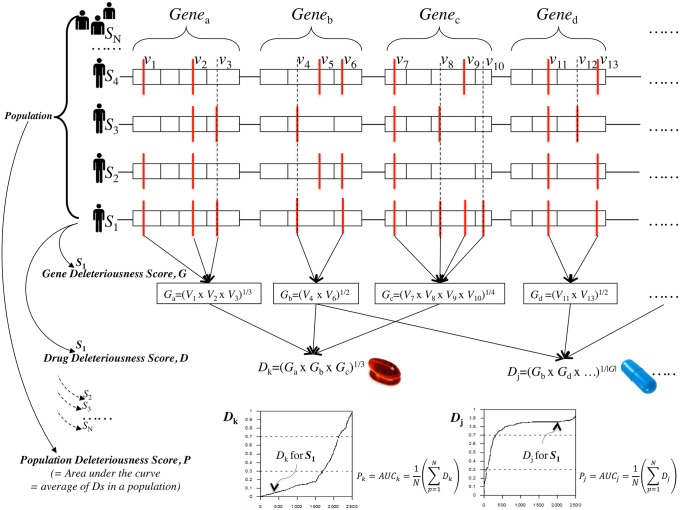
Deleteriousness scores for genes, variants, drugs, and populations. The variant score, (*V)* is the SIFT score of the variant, the gene deleteriousness score, (*G)* is the geometric mean of the *V* scores of the gene, and the drug deleteriousness score (*D*) is the geometric mean of the *G* scores for all drug-related PK/PD genes. The drug-gene relationships were obtained from the DrugBank database. The population deleteriousness score (*P*) or the area under the curve (AUC) for the distribution of the *D* score for a drug *k* in a population was computed by averaging personalized *D* scores of the entire population.

For our analysis, we used the SIFT algorithm [26] to compute the *variant* deleteriousness score (*V*) for evaluating the impact of all nonsynonymous coding variants found in the 2,807 PK/PD genes of the 2,504 personal genomes selected from the 1000 Genomes Project. The lower the SIFT score (range, 0~1) of a variant, the more deleterious the impact of the variant on the function of the gene. The *gene* deleteriousness score (*G*) of a gene was defined as the geometric mean of the *V* scores for all nonsynonymous coding variants of the gene to evaluate the overall impact of multiple deleterious variants on the gene (Fig 2). Similarly, the *drug* deleteriousness score (*D*) was defined as the geometric mean of the *G* scores for all drug-related PK/PD genes (Fig 2). All PK/PD genes of a drug were equally weighted without considering pharmacokinetic parameters. The geometric mean or the *n*th root of the product of the numbers, where *n* is the number count, was used to identify the central tendency in the change of the analyzed parameters. The *V*, *G*, and *D* deleteriousness scores ranged from 0 to 1; low *V* and *G* scores indicated severely altered structure and/or function of the corresponding gene, while low *D* value indicated increased predisposition to unintended drug responses.

Fig 2 (low panels) shows the distributions of the *D* scores for each drug (i.e., *D*_*k*_, *D*_*j*_, etc.) among the 2504 personal genomes. The *population* deleteriousness score (*P*) represented by the area under the curve (AUC) for the distribution of the *D* score for a drug *k* in a population can be computed by averaging personalized *D* scores of the entire population (Fig 2):

### Statistical Analysis

**Fig 3:**
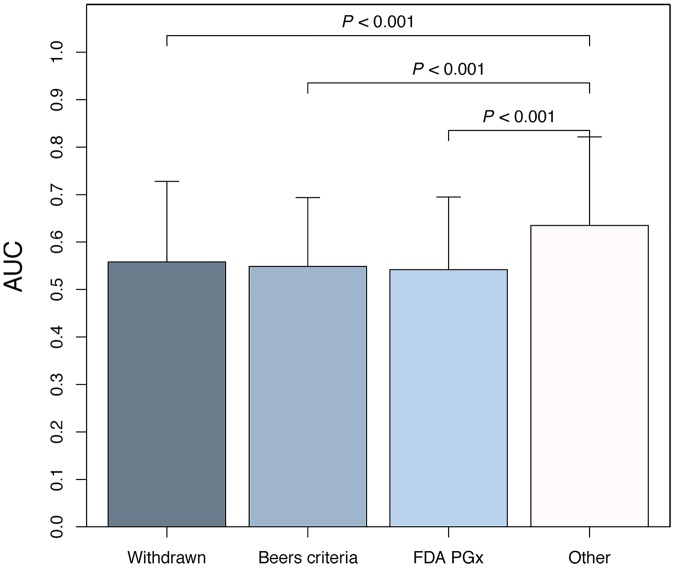
Comparison of population deleteriousness scores between withdrawn, precautionary, and other drugs. Drugs withdrawn from the market (AUC = 0.558 ±0.170), precautionary drugs according to the Beers criteria (AUC = 0.549 ±0.153), and drugs labeled by the US FDA with phamacogenomic information (AUC = 0.542 ±0.145) exhibited significantly lower AUC values than other drugs (AUC = 0.635 ±0.187; *P* < 0.001, one-way ANOVA followed by post-hoc Tukey-tests). In contrast, the difference between the three withdrawn/precautionary drug groups did not reach statistical significance (*P* > 0.05). AUC, area under the drug deleteriousness score curve; FDA PGx, FDA-approved drugs with pharmacogenomic information on drug labels.

**Fig 4:**
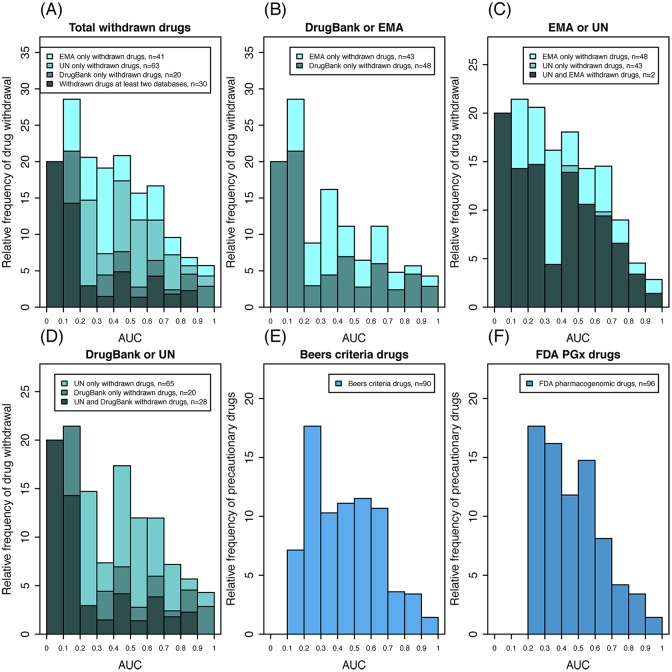
Frequency distribution of drug withdrawals and precautions according to population deleteriousness scores. The relative frequency of drug withdrawals was obtained for each of the 10 AUC score bins of equal sizes. (A) All three resources; (B–D) paired resources. Drugs with lower AUC values were more frequently withdrawn from the market (see Table 1). Relative frequencies of drug precautions according to the Beers criteria (E) and US FDA pharmacogenomics labels (F) were significantly higher in lower AUC score bins compared to higher AUC score bins (*P* < 0.01, Cochran-Armitage Trend test; see Table 1). AUC, area under the drug deleteriousness score curve; EMA, European Medicines Agency; UN, United Nations.

**Fig 5:**
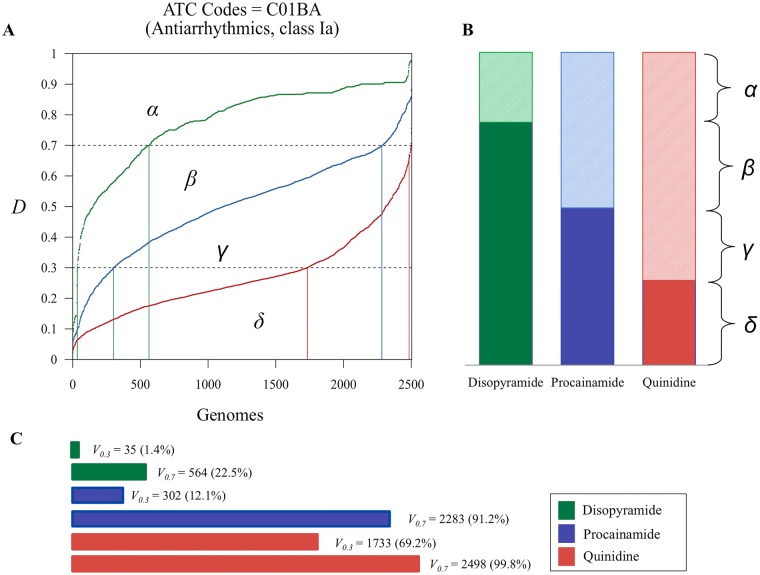
Proposed measures of drug safety to prevent precautions and drug withdrawals from the market. (A) Drug deleteriousness scores for three drugs with the same ATC code (C01BA for antiarrythmics class Ia) [disopyramide (green), procainamide (blue), and quinidine (red)] were computed for 2504 genomes from the 1000 Genomes Project and sorted in the ascending order. (B) The AUC and 1-AUC values represent population deleteriousness and genomic variability of drug-related genes, respectively. (C) When the risk threshold is set at 0.3, 35 (1.4%), 302 (12.1%), and 1733 (69.2%) individuals of the 2504 can be classified as genetically vulnerable to side effects of disopyramide, procainamide, and, quinidine respectively (*V*_0.3_). Abbreviations: AUC, area under the drug deleteriousness score curve; ATC, Anatomical Therapeutic Chemical Classification System.

ANOVA and post-hoc Tukey analysis were used to compare the AUCs among the four drug categories: withdrawn, precautionary by Beers criteria, precautionary by FDA pharmacogenomics labeling, and other drugs (Fig 3). Relative frequencies of drug withdrawals (Fig 4) and precautions (Fig 5) were computed across AUC score bins for further analysis. The Cochrane-Armitage Trend test was performed across AUC score bins to estimate the effect of the AUC scores on the frequencies of drug withdrawal and precaution (Table 1). All *P* values were two sided, and considered statistically significance at < 0.05. All statistical analyses were conducted using the R statistical package (ver. 3.01).[31]

**Table 1:** Cochran-Armitage test for trend for drug withdrawals and precautions.

AUC score bins	~0.1	~0.2	~0.3	~0.4	~0.5	~0.6	~0.7	~0.8	~0.9	~1.0	Sum.	*P* ^a^
**No. of Withdrawn Drugs**												
Total	1	4	7	13	30	34	39	16	6	4	154	<0.001
UN or EMA	1	3	7	11	26	31	34	15	4	2	134	0.001
UN or DrugBank	1	3	5	5	25	26	28	12	5	3	113	<0.001
EMA or DrugBank.	1	4	3	11	16	14	26	8	5	3	91	0.001
UN	1	2	5	3	21	23	23	11	3	1	93	0.053
EMA	0	1	2	8	6	8	12	4	1	1	43	0.001
DrugBank	1	3	1	3	10	6	14	4	4	2	48	0.007
**No. of Precautionary Drugs**												
Beers criteria	0	1	6	7	16	25	25	6	3	1	90	<0.001
FDA pharmacogenomics	0	0	6	11	17	32	19	7	3	1	96	<0.001
**No. of Total Study Drugs**	5	14	34	68	144	217	234	167	88	70	1041	

^a^*P*-values by Cochran-Armitage test for trend. Abbreviations: AUC, Area under the drug-wise deleteriousness-score curve; UN, the United Nations; EMA, the European Medicines Agency; FDA, the United States Food and Drug Administration

## Results

### Drug Withdrawals and Precautions

Altogether, we analyzed 1041 drugs from the DrugBank that had at least five listed PK/PD genes (Fig 1). Overall, 154 (14.8%) of the 1041 analyzed drugs were eventually withdrawn from the market by at least one country. Among the 137 drugs in the Beers list and 148 drugs with FDA pharmacogenomic labels, 90 (65.7%) and 96 (64.9%), respectively, met our inclusion criteria of having at least five known PK/PD genes. Interestingly, 30 (18.2%) of these precautionary drugs were also withdrawn from the market by at least one country (Fig 1, Venn diagram).

### The P Score and Drug Withdrawals/Precautions

Fig 3 demonstrates that drugs, which have been withdrawn (AUC = 0.558 ±0.170), listed as precautionary according to Beers criteria (AUC = 0.549 ±0.153), and pharmacogenomically labeled by the US FDA (AUC = 0.542 ±0.145) exhibited significantly lower AUCs compared to other drugs (AUC = 0.635 ±0.187) as indicated by ANOVA with post-hoc Tukey analysis (*P* < 0.001). As shown in S1 Fig and S1 Table, it was consistently confirmed at all thresholds of the number of PK/PD genes from 1 to 10 for study drug inclusion that the AUC values of withdrawn and precautionary drugs were significantly lower than that of other drugs (P < 0.001 by ANOVA).

Fig 4 consists of histograms that display the frequencies of drug withdrawal relative to the *population* deleteriousness score (*P*) or AUC of the drug according to the UN Consolidated Lists,[18–21] EMA,[22] and DrugBank.[16] (S2 Fig) Because each country has different policies regarding the approval of pharmaceutical products, we analyzed these resources separately as well as in combination. The results indicated that lower AUC values correlate significantly with higher relative frequencies of drug withdrawals from the market in every configuration (*P* < 0.05, Cochrane-Armitage Trend test; Table 1 and S1 Fig). The same trend was consistently observed for precautionary drugs within the Beers criteria (*P* = 0.009; Fig 4(E))[23] as well as for drugs pharmacogenomically labeled by the US FDA pharmacogenomics (*P* < 0.001, Cochrane-Armitage Trend test; Fig 4(F) and Table 1).[11]

### Genomic Variability and Drug Safety

“If it were not for the great variability among individuals, medicine might as well be a science and not be an art,” said Sir William Osler.[32] The present study demonstrated unexpectedly high person-to-person variability of deleterious variants among drug-related genes. A drug might as well be ‘genetically ideal’ if the drug-related genes have no deleterious variants across the entire human population, which is rather rare. Based on the analysis of drug-related gene variability in the population, we propose here two genomic parameters to assess drug safety, AUC and *V*_*t*_, which may facilitate drug development, use, and regulation, and eventually prevent drug withdrawal.

Fig 5 shows the distribution curve of the *D* scores among 2504 individuals for three drugs (disopyramide, procainamide, and quinidine) that belong to class Ia antiarrythmics (C01BA) according to the Anatomical Therapeutic Chemical (ATC) classification system. The AUC for disopyramide (*β*+*γ*+*δ*) is larger than that of procainamide (*γ+δ*) and quinidine (*δ*) but smaller than that of a ‘genetically ideal’ drug (*α*+*β*+*γ*+*δ*), which is equal to 1.0 and has zero person-to-person variability in the drug specific PK/PD genes. Thus, *α* (or 1-AUC of disopyramide) represents the distance of disopyramide from a ‘genetically ideal’ drug in terms of genome sequence variation. Interestingly, 1-AUC of disopyramide is smaller than that of procainamide (1-[γ+δ]) and quinidine (1-*δ*; Fig 5) indicating that among the 2504 individuals in the 1000 Genomes Project, the genes related to PK/PD of disopyramide have less deleterious variants than those of procainamide and quinidine.

Fig 5 also demonstrates that among the 2504 individuals, 35 (1.4%), 302 (12.1%), and 1733 (69.2%) persons have increased risks for ADRs caused by disopyramide, procainamide, and quinidine, respectively, if we assume that 0.3 (or 0.7) is the risk threshold for the *D* score (*V*_0.3_ (or *V*_0.7_)). *V*_t_ is defined as the proportion of people who have *D* scores lower than the risk threshold (*t*) and, therefore, are genetically vulnerable to ADRs from the use of a certain drug. The distributions of *V*_0.3_ and *V*_0.7_ for the three drugs are shown in Fig 5.

## Discussion

The decision process of drug withdrawals is highly complex and is influenced by political, commercial, ethical, and other factors. In this study, we have demonstrated that the person-to-person genome variability is a strong independent prognostic factor for drug withdrawals from the pharmaceutical market, which has long been considered unpredictable. Therefore, we propose the use of these novel measures of drug safety assessment based on personal genome sequence analysis to improve drug development, use, and regulation. The proposed method may be considered for future application in the clinical setting to identify individuals with increased genetic vulnerability to adverse side effects from a specific drug. When clinically validated for specific drugs and ADRs, healthcare providers can use this method as a tool to support clinical decisions and prevent unintended drug reactions. For pharmaceutical companies, the proposed genomic measures can be used for efficient screening of candidate pharmaceuticals, development of safer drugs, genome-based pharmacovigilance, and regulation of drug safety.

Critics have argued that clinical trials involving up to 2000~3000 participants cannot reliably detect rare ADRs with an incidence of less than 1 per 10,000.[33] Although increasing the number of trial participants is an option, albeit costly, it may still be inadequate to address the complexity of personal genome sequence variations.[34] The current affected-versus-unaffected case-control approach used in pharmacogenomics research [35] cannot provide comprehensive analysis of multiple complex associations between numerous genotypes and drugs. In contrast, the proposed *ab initio* method is scalable to increasing numbers of genotypes and drugs and relies only on integrated public resources: personal genome sequence databases covering different ethnic groups, drug PK/PD information, and the records of withdrawn and/or precautionary drugs.

Recently, US FDA recommended incorporating the ethnic category of participants in clinical trials.[36] Incorporating ethnicity-related data regarding genome sequence variability, which can affect drug safety, may be necessary for future clinical trial design. For example, to reliably detect ADRs before marketing, drugs with low AUC values (i.e., large genomic distance from a ‘genetically ideal’ drug) should be tested in larger populations than drugs with high AUC values. Considering the distribution of genetically vulnerable subpopulations or ethnic groups for individual drugs based on genome sequence variability may lead to a more rational clinical trial design and ultimately improve drug safety.

For the purpose of comprehensive evaluation, the present study was replicated with other variant function-prediction algorithms. ANNOVAR [37] provides with seven variant function prediction scores (i.e., SIFT [26], PolyPhen HIVD [27], PolyPhen HVAR [27], LRT [38], MutationTaster [28], MutationAssessor [39], FATHMM [40]) and three variant conservation scores (i.e., GERP++[41], phyloP [42], Siphy [43]). Of these 10 methods, MutationAssessor, FATHMM, and Siphy were excluded for further analysis due to their severely right-skewed variant score distributions, resulting no drug remaining in the lower score bins for the drugs with the lowest deleteriousness scores. LRT was also excluded because its binary weighting scheme of variant scores based on dN/dS ratio. As shown in S2 Table, all six prediction scores consistantely confirmed that the AUC values of withdrawn and precautionary drugs were statistically significantly lower than those of other drugs (*P* value <0.001 by ANOVA, post-hoc Tukey test). More importantly, drug withdrawal rates significantly increased as the AUC scores decreased for all six scores (*P* value < 0.05, Cochrane-Armitage tests for trend, S2 Table).

The computation of *D* scores conducted in our study is subject to some limitations due to the imperfect knowledge about the PK/PD of drugs [16] and the precise effects of nonsynonymous coding variants on protein structure and function.[26–30] The scope and accuracy of the *D* scores are also limited by insufficiency of public resources such as those that provide personal genome sequence data from various ethnic groups and standardized reports of withdrawn and precautionary drugs. Further development of these critical resources could, therefore, greatly improve and refine drug safety strategies. The proposed method has some limitations. First, the current knowledge on PK/PD drug-gene relationships is not perfect. We discovered that many drugs eligible for inclusion in our study lacked PK/PD information. Second, the current method assigns the same scores to different drugs having the same PK/PD genes without considering different pharmacokinetic parameters such as Km/Vmax. We found that less than 10% of all DrugBank drugs have at least one properly matched pharmaokinetic parameters after systematic survey of the current databases including PubChem [44], BRENDA [45], SABIO-RK [46] and MetaCyc [47]. Moreover, it may not be true for the drugs with the same PK/PD genes to have the same genetic effect in vivo because multiple factors other than genetic variants such as PK (i.e., route of administation, dose, and form), PD (i.e., targets and their physiological roles), and patient (i.e., disease, behavior, and environment) factors will affect toxicity and efficacy. Thus, given the substantial effects and utmost importance of genomic variability on drug responses, the availability of more accurate and comprehensive PK/PD data could greatly enhance drug development, use, and regulation. Third, since noncoding regions of the human genome contain up to 88% of the weakly phenotype-associated variants as identified by the GWAS [48] and ENCODE (ENCyclopedia Of DNA Elements)[49] the integration of both coding and noncoding gene information could further improve the present scoring system.

To the best of our knowledge, this is the first study that has addressed the long-standing problem of unpredictability in pharmaceutical market withdrawals by incorporating state-of-the-art personal genome sequencing technology. The strong correlation between the population deleteriousness score (or AUC) and pharmaceutical market withdrawal rate was consistent among different databases and lists of drug withdrawals and precautions, including UN Consolidated Lists, DrugBank, European Medicines Agency, Beers guidelines, and US FDA. One interesting byproduct of our study is that the proposed method may rescue necessary drugs that have already been withdrawn from the market for safety reasons [8]. Clinical reinstatement of a previously withdrawn drug, however, should be deferred until the genetic cause of ADRs in a vulnerable subpopulation has been clearly identified and corresponding diagnostic tests have been developed.

## Supporting Information

S1 Fig Comparison of population deleteriousness scores between withdrawn, precautionary, and other drugs across different numbers of drug-related genes. Population deleteriousness scores (AUC) were significantly lower for the three withdrawn and precautionary drug groups than others at all thresholds (i.e., the numbers of PK/PD genes from 1 to 10) (P < 0.05 by post-hoc Tukey tests after one-way ANOVA (P < 0.001)) for study drug inclusion. In contrast, population deleteriousness scores did not show statistically significant difference among the three withdrawn and precautionary drug groups at all thresholds (*P* > 0.05). **P < 0.001 and * *P* < 0.05 by post-hoc Tukey test (see Table 1). Numbers in parentheses represent the numbers of included drugs at each threshold. AUC, area under the drug deleteriousness score curve; FDA PGx, FDA-approved drugs with pharmacogenomic information on drug labels; PD, Pharmacodynamics; PK, Pharmacokinetics.
(DOCX)

S2 Fig Drug and frequency distribution of drug withdrawals from the UN, DrugBank and EMA according to population deleteriousness scores. The relative frequency of drug withdrawals was obtained for each of the 10 AUC score bins of equal sizes from the three individual databases and all combination; (A) Total withdrawn from three different resources, (B) UN, (C) DrugBank and (D) EMA. The three icons in A (lower panel) show drug deleteriousness score curves typical for the corresponding AUC score bins. The shaded area representing 1-AUC shows the distance of the drug from a ‘genetically ideal’ pharmaceutical in terms of genome sequence variation, i.e., no variation of relevant genes between individuals. AUC, area under the drug deleteriousness score curve; EMA, European Medicines Agency; UN, United Nations.
(TIFF)

S1 File 1041 including drugs with five or more pharmacokinetics/pharmacodynamic genes. Among 5099 drugs had at least one identified PK/PD genes from the DrugBank Version 4.1, excluding 4058 drugs having less than five PK/PD genes, 1041 drugs having five or more PK/PD genes were included. For these inclusion drugs, we matched the drug regulation information for further analysis.
(XLSX)

S2 File Total withdrawn and precautionary drugs. Withdrawn or precautionary drug information was collected comprehensively from the multiple publicly available resources. For withdrawn drugs, we reviewed reports from United Nations, European Medicines Agency, and DrugBank annotation. For precautionary drugs, we collected information from the Beers criteria and US FDA pharmacogenomic biomarker information in drug labels.
(XLSX)

S1 Table Comparison of population deleteriousness scores between withdrawn, precautionary, and other drugs at all thresholds of different number of PK/PD genes for drug inclusion. * P < 0.05 and ** P < 0.001 by post-hoc Tukey test after one-way ANOVA in comparison to other drugs. † P < 0.001 by one-way ANOVA at all thresholds of different numbers of drugs for study drug inclusion. ‡ P > 0.05 by post-hoc Tukey test after one-way ANOVA for all pairwise comparisons between the the three drug groups that are withdrawn and precautionary. Population score values are mean (SD) (see S1 Fig.) AUC, area under the drug deleteriousness score curve; FDA PGx, FDA-approved drugs with pharmacogenomic information on drug labels; PD, Pharmacodynamics; PK, Pharmacokinetics; P score, Population deleteriousness score.
(DOCX)

S2 Table Descriptive statistics and statistical test results for six function prediction scores. For each score, all including drugs were collected independently. We included drugs with at least five identified pharmacokinetics (PK) and pharmacodynamics (PD) gene relationships (which have specific score annotated variants) in the analysis. * *P* < 0.05 and ** *P* < 0.001. AUC, area under the drug deleteriousness score curve; EMA, European Medicines Agency; FDA PGx, FDA-approved drugs with pharmacogenomic information on drug labels; UN, United Nations.
(DOCX)

## References

[1] Ma Q, Lu AY. Pharmacogenetics, pharmacogenomics, and individualized medicine. Pharmacol Rev. 2011;63(2):437–59. 10.1124/pr.110.003533 21436344

[2] Wysowski DK, Swartz L. Adverse drug event surveillance and drug withdrawals in the United States, 1969–2002: the importance of reporting suspected reactions. Arch Intern Med. 2005;165(12):1363–9. 10.1001/archinte.165.12.1363 15983284

[3] Lazarou J, Pomeranz BH, Corey PN. Incidence of adverse drug reactions in hospitalized patients: a meta-analysis of prospective studies. JAMA. 1998;279(15):1200–5. 10.1001/jama.279.15.1200 9555760

[4] Wilkinson GR. Drug metabolism and variability among patients in drug response. N Engl J Med. 2005;352(21):2211–21. 10.1056/NEJMra032424 15917386

[5] Lasser KE, Allen PD, Woolhandler SJ, Himmelstein DU, Wolfe SM, Bor DH. Timing of new black box warnings and withdrawals for prescription medications. JAMA. 2002;287(17):2215–20. 10.1001/jama.287.17.2215 11980521

[6] Ninan B, Wertheimer AI. Withdrawing Drugs in the U.S. Versus Other Countries. Inov Pharm. 2012;3(87):1–12.22844651

[7] Lexchin J. How Safe Are New Drugs? Market Withdrawal of Drugs Approved in Canada between 1990 and 2009. Open Med. 2014;8(1):e14–e19. 25009681 PMC4085091

[8] Shah RR. Can pharmacogenetics help rescue drugs withdrawn from the market? Pharmacogenomics. 2006;7(6):889–908. 10.2217/14622416.7.6.889 16981848

[9] Daggumalli JSV, Martin IG. Are Pharmaceutical Market Withdrawals Preventable? A Preliminary Analysis. Drug Inf J. 2012;46:694–700.

[10] Wang L, McLeod HL, Weinshilboum RM. Genomics and drug response. N Engl J Med. 2011;364(12):1144–53. 10.1056/NEJMra1010600 21428770 PMC3184612

[11] U.S. Food and Drug Administration. Table of Pharmacogenomic Biomarkers in Drug Labeling. 2015. [cited 2015 September 8]. In: U.S. FDA Site [Internet]. Maryland. Available: http://www.fda.gov/drugs/scienceresearch/researchareas/pharmacogenetics/ucm083378.htm

[12] MacArthur DG, Balasubramanian S, Frankish A, Huang N, Morris J, Walter K, et al A systematic survey of loss-of-function variants in human protein-coding genes. Science. 2012;335(6070):823–8. 10.1126/science.1215040 22344438 PMC3299548

[13] Roden DM, George AL Jr. The genetic basis of variability in drug responses. Nat Rev Drug Discov. 2002;1(1):37–44. 10.1038/nrd705 12119608

[14] Genomes Project C, Auton A, Brooks LD, Durbin RM, Garrison EP, Kang HM, et al A global reference for human genetic variation. Nature. 2015;526(7571):68–74. 10.1038/nature15393 26432245 PMC4750478

[15] Heiat A, Gross CP, Krumholz HM. Representation of the elderly, women, and minorities in heart failure clinical trials. Arch Intern Med. 2002;162(15):1682–8. 10.1001/archinte.162.15.1682 12153370

[16] Wishart DS, Knox C, Guo AC, Shrivastava S, Hassanali M, Stothard P, et al DrugBank: a comprehensive resource for in silico drug discovery and exploration. Nucleic Acids Res. 2006;34(Database issue):D668–72. 10.1093/nar/gkj067 16381955 PMC1347430

[17] Genomes Project C, Abecasis GR, Auton A, Brooks LD, DePristo MA, Durbin RM, et al An integrated map of genetic variation from 1,092 human genomes. Nature. 2012;491(7422):56–65. 10.1038/nature11632 23128226 PMC3498066

[18] Consolidated List of Products Whose Consumption and/or Sale Have Been Banned, Withdrawn, Severely Restricted or not Approved by Governments: Pharmaceuticals. Eighth Issue. New York: United Nation; 2003.

[19] Consolidated List of Products Whose Consumption and/or Sale Have Been Banned, Withdrawn, Severely Restricted or not Approved by Governments: Pharmaceuticals. Tenth Issue. New York: United Nation; 2004.

[20] Consolidated List of Products Whose Consumption and/or Sale Have Been Banned, Withdrawn, Severely Restricted or not Approved by Governments: Pharmaceuticals. Twelfth Issue. New York: United Nation; 2005.

[21] Consolidated List of Products Whose Consumption and/or Sale Have Been Banned, Withdrawn, Severely Restricted or not Approved by Governments: Pharmaceuticals. Fourteenth Issue. New York: United Nation; 2009

[22] Annual report of the European Medicines Agency [Internet]. European Medicines Agency; 2009 [cited 2015 September 8]. Available: http://www.ema.europa.eu/docs/en_GB/document_library/Annual_report/2010/05/WC500090712.pdf

[23] By the American Geriatrics Society Beers Criteria Update Expert P. American Geriatrics Society 2015 Updated Beers Criteria for Potentially Inappropriate Medication Use in Older Adults. J Am Geriatr Soc. 2015;63(11):2227–46. 10.1111/jgs.13702 26446832

[24] Beers MH, Ouslander JG, Rollingher I, Reuben DB, Brooks J, Beck JC. Explicit criteria for determining inappropriate medication use in nursing home residents. Arch Intern Med. 1991;151(9):1825–32. 10.1001/archinte.1991.00400090107019 1888249

[25] Campanelli CM. American Geriatrics Society updated beers criteria for potentially inappropriate medication use in older adults: the American Geriatrics Society 2012 Beers Criteria Update Expert Panel. J Am Geriatr Soc. 2012;60(4):616 10.1111/j.1532-5415.2012.03923.x 22376048 PMC3571677

[26] Kumar P, Henikoff S, Ng PC. Predicting the effects of coding non-synonymous variants on protein function using the SIFT algorithm. Nat Protoc. 2009;4(7):1073–81. Epub 2009/06/30. 10.1038/nprot.2009.86 19561590

[27] Ramensky V, Bork P, Sunyaev S. Human non‐synonymous SNPs: server and survey. Nucleic Acids Res. 2002;30(17):3894–900. 10.1093/nar/gkf493 12202775 PMC137415

[28] Reva B, Antipin Y, Sander C. Determinants of protein function revealed by combinatorial entropy optimization. Genome Biol. 2007;8(11):R232 Epub 2007/11/03. 10.1186/gb-2007-8-11-r232 17976239 PMC2258190

[29] Kircher M, Witten DM, Jain P, O’Roak BJ, Cooper GM, Shendure J. A general framework for estimating the relative pathogenicity of human genetic variants. Nat Genet. 2014;46(3):310 10.1038/ng.2892 24487276 PMC3992975

[30] González-Pérez A, López-Bigas N. Improving the assessment of the outcome of nonsynonymous SNVs with a consensus deleteriousness score, Condel. Am J Hum Genet. 2011;88(4):440–9. 10.1016/j.ajhg.2011.03.004 21457909 PMC3071923

[31] Team. RDC. R: A language and environment for statistical computing. Vienna, Austria: R Foundation for statistical Computing Retrieved from http://www.R-project.org; 2010.

[32] Osler W. The principles and practice of medicine: New York: Appleton; 1909 10.2307/3403146

[33] Strom BL. How the US drug safety system should be changed. JAMA. 2006;295(17):2072–5. 10.1001/jama.295.17.2072 16670415

[34] Berlin JA, Glasser SC, Ellenberg SS. Adverse event detection in drug development: recommendations and obligations beyond phase 3. Am J Public Health. 2008;98(8):1366–71. 10.2105/AJPH.2007.124537 18556607 PMC2446471

[35] Abernethy DR, Woodcock J, Lesko LJ. Pharmacological mechanism-based drug safety assessment and prediction. Clin Pharmacol Ther. 2011;89(6):793–7. 10.1038/clpt.2011.55 21490594

[36] Guidance for Industry: Collection of Race and Ethnicity Data in Clinical Trials [Internet]. US Food and Drug Administration. [cited 2015 September 8]. In: U.S. FDA webpage. Rockville, Maryland. Available:. http://www.fda.gov/downloads/RegulatoryInformation/Guidances/ucm126396.pdf

[37] Wang K, Li M, Hakonarson H. ANNOVAR: functional annotation of genetic variants from high-throughput sequencing data. Nucleic Acids Res. 2010;38(16):e164–e. 10.1093/nar/gkq603 20601685 PMC2938201

[38] Chun S, Fay JC. Identification of deleterious mutations within three human genomes. Genome Res. 2009;19(9):1553–61. 10.1101/gr.092619.109 19602639 PMC2752137

[39] Cooper GM, Goode DL, Ng SB, Sidow A, Bamshad MJ, Shendure J, et al Single-nucleotide evolutionary constraint scores highlight disease-causing mutations. Nat Methods. 2010;7(4):250–1. 10.1038/nmeth0410-250 20354513 PMC3145250

[40] Shihab HA, Gough J, Cooper DN, Day IN, Gaunt TR. Predicting the functional consequences of cancer-associated amino acid substitutions. Bioinformatics. 2013:btt182 10.1093/bioinformatics/btt182 23620363 PMC3673218

[41] Davydov EV, Goode DL, Sirota M, Cooper GM, Sidow A, Batzoglou S. Identifying a high fraction of the human genome to be under selective constraint using GERP++. PLoS Comput Biol. 2010;6(12):e1001025 10.1371/journal.pcbi.1001025 21152010 PMC2996323

[42] Pollard KS, Hubisz MJ, Rosenbloom KR, Siepel A. Detection of nonneutral substitution rates on mammalian phylogenies. Genome Res. 2010;20(1):110–21. 10.1101/gr.097857.109 19858363 PMC2798823

[43] Garber M, Guttman M, Clamp M, Zody MC, Friedman N, Xie X. Identifying novel constrained elements by exploiting biased substitution patterns. Bioinformatics. 2009;25(12):i54–i62. 10.1093/bioinformatics/btp190 19478016 PMC2687944

[44] Kim S, Thiessen PA, Bolton EE, Chen J, Fu G, Gindulyte A, et al PubChem substance and compound databases. Nucleic Acids Res. 2015; 4; 44(Database issue): D1202–D1213. 10.1093/nar/gkv951 26400175 PMC4702940

[45] Schomburg I, Chang A, Schomburg D. BRENDA, enzyme data and metabolic information. Nucleic Acids Res. 2002;30(1):47–9. 10.1016/S0968-0004(01)02027-8 11752250 PMC99121

[46] Wittig U, Kania R, Golebiewski M, Rey M, Shi L, Jong L, et al SABIO-RK—database for biochemical reaction kinetics. Nucleic Acids Res. 2012; 40(Database issue): D790–D796. 10.1093/nar/gkr1046 22102587 PMC3245076

[47] Caspi R, Altman T, Dale JM, Dreher K, Fulcher CA, Gilham F, et al The MetaCyc database of metabolic pathways and enzymes and the BioCyc collection of pathway/genome databases. Nucleic Acids Res. 2010; 4; 44(Database issue): D471–D480. 10.1093/nar/gkv1164 26527732 PMC4702838

[48] Schaub MA, Boyle AP, Kundaje A, Batzoglou S, Snyder M. Linking disease associations with regulatory information in the human genome. Genome Res. 2012;22(9):1748–59. 10.1101/gr.136127.111 22955986 PMC3431491

[49] Consortium EP. The ENCODE (ENCyclopedia Of DNA Elements) Project. Science. 2004;306(5696):636–40. 10.1126/science.1105136. 15499007

