# Supplementary material for: Genome Sequence Variability Predicts Drug Precautions and Withdrawals from the Market
Source: PLoS One. 2016 Sep 30;11(9):e0162135. doi: 10.1371/journal.pone.0162135 (PMC5045182; doi:10.1371/journal.pone.0162135)
Supplement: S1 Fig — Population deleteriousness scores (AUC) were significantly lower for the three withdrawn and precautionary drug groups than others at all thresholds (i.e., the numbers of PK/PD genes from 1 to 10) (P < 0.05 by post-hoc Tukey tests after one-way ANOVA (P < 0.001)) for study drug inclusion. In contrast, population deleteriousness scores did not show statistically significant difference among the three withdrawn and precautionary drug groups at all thresholds (P > 0.05). **P < 0.001 and * P < 0.05 by post-hoc Tukey test (see Table 1). Numbers in parentheses represent the numbers of included drugs at each threshold. AUC, area under the drug deleteriousness score curve; FDA PGx, FDA-approved drugs with pharmacogenomic information on drug labels; PD, Pharmacodynamics; PK, Pharmacokinetics. (DOCX) [file pone.0162135.s001.docx]

**Supplementary Figure 1. Comparison of population deleteriousness scores between withdrawn, precautionary, and other drugs across different numbers of drug-related genes**

Population deleteriousness scores (AUC) were significantly lower for the three withdrawn and precautionary drug groups than others at all thresholds (i.e., the numbers of PK/PD genes from 1 to 10) (*P* < 0.05 by post-hoc Tukey tests after one-way ANOVA (*P* < 0.001)) for study drug inclusion. In contrast, population deleteriousness scores did not show statistically significant difference among the three withdrawn and precautionary drug groups at all thresholds (*P* > 0.05). ***P* < 0.001 and * *P* < 0.05 by post-hoc Tukey test (see eTable 1). Numbers in parentheses represent the numbers of included drugs at each threshold. AUC, area under the drug deleteriousness score curve; FDA PGx, FDA-approved drugs with pharmacogenomic information on drug labels; PD, Pharmacodynamics; PK, Pharmacokinetics.
