# Supplementary figures and images for: Genome Sequence Variability Predicts Drug Precautions and Withdrawals from the Market
Source: PLoS One. 2016 Sep 30;11(9):e0162135. doi: 10.1371/journal.pone.0162135 (PMC5045182; doi:10.1371/journal.pone.0162135)

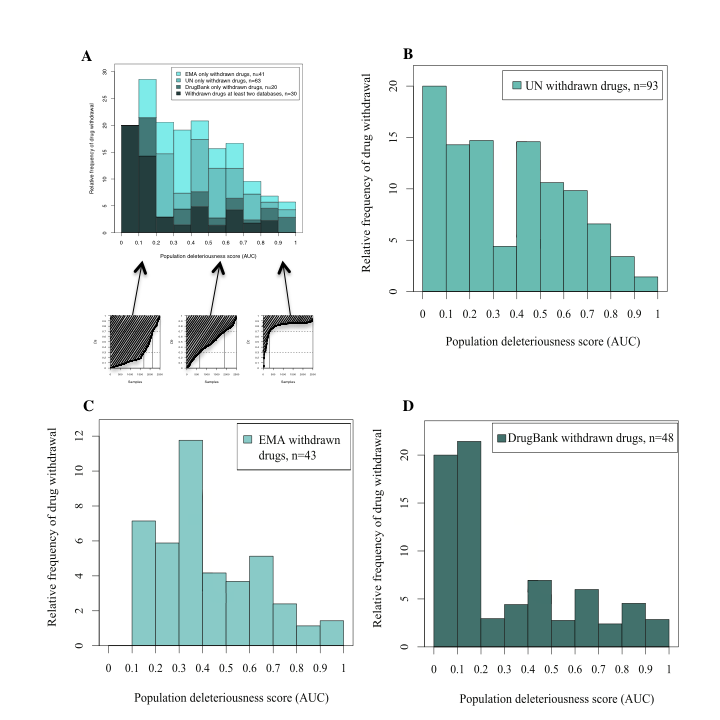

Supplement: S2 Fig — The relative frequency of drug withdrawals was obtained for each of the 10 AUC score bins of equal sizes from the three individual databases and all combination; (A) Total withdrawn from three different resources, (B) UN, (C) DrugBank and (D) EMA. The three icons in A (lower panel) show drug deleteriousness score curves typical for the corresponding AUC score bins. The shaded area representing 1-AUC shows the distance of the drug from a ‘genetically ideal’ pharmaceutical in terms of genome sequence variation, i.e., no variation of relevant genes between individuals. AUC, area under the drug deleteriousness score curve; EMA, European Medicines Agency; UN, United Nations. (TIFF) [file pone.0162135.s002.tiff]
