# Supplementary material for: Genome Sequence Variability Predicts Drug Precautions and Withdrawals from the Market
Source: PLoS One. 2016 Sep 30;11(9):e0162135. doi: 10.1371/journal.pone.0162135 (PMC5045182; doi:10.1371/journal.pone.0162135)
Supplement: S1 Table — * P < 0.05 and ** P < 0.001 by post-hoc Tukey test after one-way ANOVA in comparison to other drugs. † P < 0.001 by one-way ANOVA at all thresholds of different numbers of drugs for study drug inclusion. ‡ P > 0.05 by post-hoc Tukey test after one-way ANOVA for all pairwise comparisons between the the three drug groups that are withdrawn and precautionary. Population score values are mean (SD) (see S1 Fig.) AUC, area under the drug deleteriousness score curve; FDA PGx, FDA-approved drugs with pharmacogenomic information on drug labels; PD, Pharmacodynamics; PK, Pharmacokinetics; P score, Population deleteriousness score. (DOCX) [file pone.0162135.s005.docx]

**Supplementary Table 1. Comparison of population deleteriousness scores between withdrawn, precautionary, and other drugs at all thresholds of different number of PK/PD genes for drug inclusion**

| No. of PK/PD Genes^†^ | Withdrawn and precautionary drugs^‡^ | | | | | | | | | | |  | Other drugs | |
| --- | --- | --- | --- | --- | --- | --- | --- | --- | --- | --- | --- | --- | --- | --- |
|  | Withdrawn | | |  | Beers criteria | | |  | FDA PGx | | |  |  |  |
|  | No. of drugs | *P* score (AUC) |  |  | No. of drugs | *P* score (AUC) |  |  | No. of drugs | *P* score (AUC) |  |  | No. of drugs | *P* score (AUC) |
| 1≤ | 270 | 0.646 (0.205) | ^**^ |  | 115 | 0.582 (0.181) | ^**^ |  | 127 | 0.589 (0.181) | ^**^ |  | 4644 | 0.768 (0.226) |
| 2≤ | 231 | 0.614 (0.190) | ^**^ |  | 110 | 0.566 (0.167) | ^**^ |  | 120 | 0.582 (0.173) | ^**^ |  | 1966 | 0.701 (0.211) |
| 3≤ | 199 | 0.595 (0.181) | ^**^ |  | 105 | 0.564 (0.170) | ^**^ |  | 116 | 0.571 (0.164) | ^**^ |  | 1264 | 0.664 (0.203) |
| 4≤ | 170 | 0.571 (0.172) | ^**^ |  | 96 | 0.552 (0.164) | ^**^ |  | 109 | 0.560 (0.156) | ^**^ |  | 947 | 0.646 (0.192) |
| 5≤ | 154 | 0.558 (0.170) | ^**^ |  | 90 | 0.549 (0.153) | ^**^ |  | 96 | 0.542 (0.145) | ^**^ |  | 752 | 0.635 (0.186) |
| 6≤ | 136 | 0.545 (0.168) | ^**^ |  | 85 | 0.536 (0.145) | ^**^ |  | 86 | 0.528 (0.138) | ^**^ |  | 603 | 0.624 (0.179) |
| 7≤ | 121 | 0.533 (0.160) | ^**^ |  | 82 | 0.530 (0.142) | ^**^ |  | 80 | 0.519 (0.136) | ^**^ |  | 498 | 0.611 (0.175) |
| 8≤ | 110 | 0.533 (0.152) | ^*^ |  | 74 | 0.528 (0.127) | ^*^ |  | 77 | 0.518 (0.131) | ^**^ |  | 421 | 0.601 (0.176) |
| 9≤ | 97 | 0.534 (0.158) | ^*^ |  | 68 | 0.528 (0.131) | ^*^ |  | 67 | 0.504 (0.131) | ^**^ |  | 357 | 0.593 (0.171) |
| 10≤ | 91 | 0.530 (0.160) | ^*^ |  | 68 | 0.528 (0.130) | ^*^ |  | 65 | 0.500 (0.130) | ^*^ |  | 310 | 0.587 (0.173) |
