# Supplementary material for: Genome Sequence Variability Predicts Drug Precautions and Withdrawals from the Market
Source: PLoS One. 2016 Sep 30;11(9):e0162135. doi: 10.1371/journal.pone.0162135 (PMC5045182; doi:10.1371/journal.pone.0162135)
Supplement: S2 Table — For each score, all including drugs were collected independently. We included drugs with at least five identified pharmacokinetics (PK) and pharmacodynamics (PD) gene relationships (which have specific score annotated variants) in the analysis. * P < 0.05 and ** P < 0.001. AUC, area under the drug deleteriousness score curve; EMA, European Medicines Agency; FDA PGx, FDA-approved drugs with pharmacogenomic information on drug labels; UN, United Nations. (DOCX) [file pone.0162135.s006.docx]

**Table S2. Descriptive statistics and statistical test results for six function prediction scores**

| Groups | | SIFT | Polyphen-HVAR | Polyphen-HIVD | MutationTaster | Phylop | GERP++ |
| --- | --- | --- | --- | --- | --- | --- | --- |
| Including drugs | Number of drugs | 1041 | 1040 | 1040 | 1040 | 1040 | 1040 |
| Withdrawn drugs | AUC mean (SD) | 0.56 (0.17) | 0.44 (0.28) | 0.51 (0.26) | 0.12 (0.23) | 0.22 (0.18) | 0.28 (0.18) |
|  | Number of drugs | 154 | 154 | 154 | 154 | 154 | 154 |
| FDA PGx drugs | AUC mean (SD) | 0.54 (0.15) | 0.34 (0.25) | 0.42 (0.24) | 0.04 (0.11) | 0.16 (0.10) | 0.23 (0.11) |
|  | Number of drugs | 96 | 96 | 96 | 96 | 96 | 96 |
| Beers criteria drugs | AUC mean (SD) | 0.55 (0.15) | 0.40 (0.27) | 0.47 (0.26) | 0.05 (0.15) | 0.17 (0.13) | 0.22 (0.13) |
|  | Number of drugs | 90 | 90 | 90 | 90 | 90 | 90 |
| Other drugs | AUC mean (SD) | 0.64 (0.19) | 0.56 (0.30) | 0.63 (0.27) | 0.20 (0.30) | 0.29 (0.25) | 0.34 (0.25) |
|  | Number of drugs | 752 | 751 | 751 | 751 | 751 | 751 |
| ANOVA^*^  Post-hoc Tukey *p* value | FDA PGx vs Beers | 0.994 | 0.561 | 0.579 | 1.000 | 0.998 | 0.995 |
|  | Withdrawn vs Beers | 0.979 | 0.689 | 0.638 | 0.154 | 0.306 | 0.182 |
|  | Withdrawn vs FDA PGx | 0.9 | 0.047 | 0.041 | 0.129 | 0.202 | 0.28 |
|  | Other vs Withdrawn* | <0.001 | <0.001 | <0.001 | 0.009 | 0.001 | 0.01 |
|  | Other vs Beers** | <0.001 | <0.001 | <0.001 | <0.001 | <0.001 | <0.001 |
|  | Other vs FDA PGx** | <0.001 | <0.001 | <0.001 | <0.001 | <0.001 | <0.001 |
| *p* value of Cochrane-Armitage test for trend acoross 10 AUC score bins | Total withdrawn* | <0.001 | <0.001 | <0.001 | 0.035 | 0.005 | 0.038 |
|  | UN or DrugBank | 0.001 | 0.057 | 0.044 | 0.286 | 0.074 | 0.171 |
|  | UN or EMA* | <0.001 | <0.001 | <0.001 | 0.022 | 0.001 | 0.009 |
|  | DrugBank or EMA | 0.001 | 0.015 | 0.017 | 0.028 | 0.056 | 0.165 |
|  | DrugBank only | 0.053 | 0.947 | 0.995 | 0.409 | 0.689 | 0.807 |
|  | UN only | 0.001 | 0.007 | 0.004 | 0.215 | 0.018 | 0.05 |
|  | EMA only | 0.007 | <0.001 | 0.001 | 0.024 | 0.022 | 0.086 |
|  | FDA PGx** | <0.001 | <0.001 | <0.001 | <0.001 | <0.001 | <0.001 |
|  | Beers criteria** | <0.001 | <0.001 | <0.001 | <0.001 | <0.001 | <0.001 |
